# Exploration of Cytokines That Impact the Therapeutic Efficacy of Mesenchymal Stem Cells in Alzheimer’s Disease

**DOI:** 10.3390/bioengineering12060646

**Published:** 2025-06-12

**Authors:** Herui Wang, Chonglin Zhong, Yi Mi, Guo Li, Chenliang Zhang, Yaoyao Chen, Xin Li, Yongjun Liu, Guangyang Liu

**Affiliations:** 1 Stem Cell Biology and Regenerative Medicine Department, Yi-Chuang Institute of Bio-Industry, Beijing 100176, China; wangheruime@163.com (H.W.);; 2 Department of Anesthesiology, Xuanwu Hospital, Capital Medical University, Beijing 100053, China

**Keywords:** Alzheimer’s disease, neuroinflammation, cognition, nerve regeneration, mesenchymal stem cells

## Abstract

Current therapies for Alzheimer’s disease (AD) includes acetylcholinesterase inhibitors, NMDA receptor antagonists, and amyloid beta (Aβ)/Tau-targeting drugs. While these drugs improve cognitive decline and target the pathological mechanisms, their outcomes still are still in debate. Mesenchymal stem cells (MSCs) offer a regenerative approach by modulating neuroinflammation and promoting neuroprotection. Although the paracrine of MSCs is efficient in various AD preclinical studies and the exosomes of MSCs have entered clinical trials, the key cytokines driving the efficacy remain unclear. Here, we evaluated human umbilical cord-derived MSCs (hUC-MSCs) and employed gene-silenced MSCs (siHGF-MSCs, siTNFR1-MSCs, siBDNF-MSCs) in APP/PS1 AD mice to investigate specific mechanisms. hUC-MSCs significantly reduced Aβ/Tau pathology and neuroinflammation, with cytokine-specific contributions: silencing HGF predominantly reduced Aβ/Tau clearance, although silencing TNFR1 or BDNF showed modest effects; silencing TNFR1 or BDNF more prominently weakened anti-neuroinflammation, while silencing HGF exerted a weaker influence. All three cytokines partially contributed to oxidative stress reduction and cognitive improvements. Our study highlights MSC-driven AD alleviation as a multifactorial strategy and reveals specific cytokines alleviating different aspects of AD pathology.

## 1. Introduction

Alzheimer’s disease (AD) is a common neurodegenerative disorder marked by gradual cognitive decline, extracellular amyloid-beta (Aβ) plaques, and intracellular neurofibrillary tangles of hyperphosphorylated Tau protein [1]. The accumulation of Aβ and Tau triggers neuroinflammation and oxidative stress, exacerbating injury and death in neural cells, ultimately causing synaptic loss and irreversible cognitive impairment [2]. Despite many years of investigation, existing therapeutic approaches for AD still face considerable challenges in their effectiveness in stopping or reversing the progression of the disease.

Acetylcholinesterase inhibitors (AChEIs) and NMDA receptor antagonists have been indicated for the treatment of AD; additionally, several anti-Aβ monoclonal antibodies and anti-Tau monoclonal antibodies have received approval from FDA for therapeutic use [3]. While AChEIs and NMDA receptor antagonists provide symptomatic relief and cognitive benefits, they do not stop or reverse the underlying progression of AD [4]. In contrast, therapies aimed at Aβ and Tau are intended to target the essential pathological processes of AD. However, despite promising results in preclinical studies, their clinical efficacy remains controversial. Factors such as the timing of intervention, medical dosage, and patient selection may contribute to the inconsistent outcomes observed in clinical trials [5]. Moreover, the development of many Aβ- or Tau-targeting medicines has been discontinued due to limited efficacy and adverse effects.

Targeting neuroinflammation and promoting neuro-regeneration represent distinct strategies to mitigate AD [6,7]. Mesenchymal stem cells (MSCs) have gained attraction as promising candidates for AD therapy due to their immunomodulatory, neuroprotective, and regenerative properties [8]. MSCs can exert therapeutic effects on various diseases via paracrine secretion [9]. In contrast to drugs that target a single pathway, the paracrine factors released by MSCs contains different classes of molecules, including miRNAs, cytokines, and growth factors, which may simultaneously modulate neuroinflammation, enhance neuronal survival, and promote Aβ and Tau clearance [10,11]. Notably, preclinical studies have demonstrated that MSC transplantation reduces Aβ burden, mitigates Tau pathology, and improves cognitive performance in AD models [8,12]. However, the exact cytokines responsible for mediating these effects and the underlying molecular mechanisms are not completely understood.

Previously, we have observed that hUC-MSCs could significantly reduce Aβ plaque and p-Tau in the entorhinal cortex (EC) and CA1 region of the hippocampus in APP/PS1 mice. Based on these results, we examined the contributions of hepatocyte growth factor (HGF), tumor necrosis factor receptor 1 (TNFR1), and brain-derived neurotrophic factor (BDNF) to the therapeutic efficacy in APP/PS1 mice. All MSCs were administered intravenously. Compared to intravenous infusion, intracerebroventricular injection avoids the entrapment of cells in the lung; however, this route poses a risk of causing neuronal injury. A phase I clinical trial reported adverse events following the intracerebroventricular administration of MSCs [13]. To mitigate the influence of adverse events on results, intravenous injection was selected. HGF and HGF-expressing hUC-MSCs can improve recognition, reduce p-Tau deposition, and alleviate neuronal injury in SAMP8 mice [14]. By applying siHGF-MSCs to the APP/PS1 model, we aim to observe whether HGF influences Aβ clearance and neuroinflammation regulation in hUC-MSCs. TNFR1 was chosen because it serves as both an inflammation propagator and inflammation inhibitor [15,16]. We have demonstrated that TNFR1 is essential for hUC-MSCs-mediated inflammatory suppression in a rheumatoid arthritis model [16]. It is crucial to determine whether it directs MSCs to exacerbate or inhibit neuronal inflammation. BDNF plays an important role in neuronal plasticity and promotes cognition. Lower levels of BDNF have been found in patients and in the 5xFAD model [17]. Overexpression of BDNF reduced inflammatory cytokines levels in a human nerve cell line [18]. The application of BDNF-overexpressing MSCs can protect cortical neurons in vitro [17]. By employing siBDNF-MSC, we aim to observe the protective effect in vivo.

## 2. Materials and Methods

### 2.1. Mouse Grouping and Drug Administration Protocol

APP/PS1 mice were randomly divided into five groups based on body weight: a control group and four treatment groups (hUC-MSC, siHGF-MSC, siTNFR1-MSC, and siBDNF-MSC), with nine mice in each group. An additional group of nine C57BL/6J mice served as the healthy control group.

Cell treatment began at eight months of age for the APP/PS1 mice. The mice were administered the cells every two weeks for a total of three doses (at weeks 0, 2, and 4). The endpoint of the experiment was six weeks after the first dose (day 42). The cells were administered via tail vein injection. Each treatment group received hUC-MSCs, siHGF-MSCs, siTNFR1-MSCs, or siBDNF-MSCs at a dose of 2 × 10^6^ cells per mouse in a volume of 0.2 mL. The healthy and control groups received an equivalent volume of saline.

The animal experiments were approved by the Laboratory Animal Ethics Committee of Youji Pharmaceutical Technology Co., Ltd., (Tianjin, China) with IACUC number: IACUC20220621-02.00. The experimental process was carried out in accordance with all animal welfare requirements.

### 2.2. Novel Object Recognition Test

**Figure 1:**
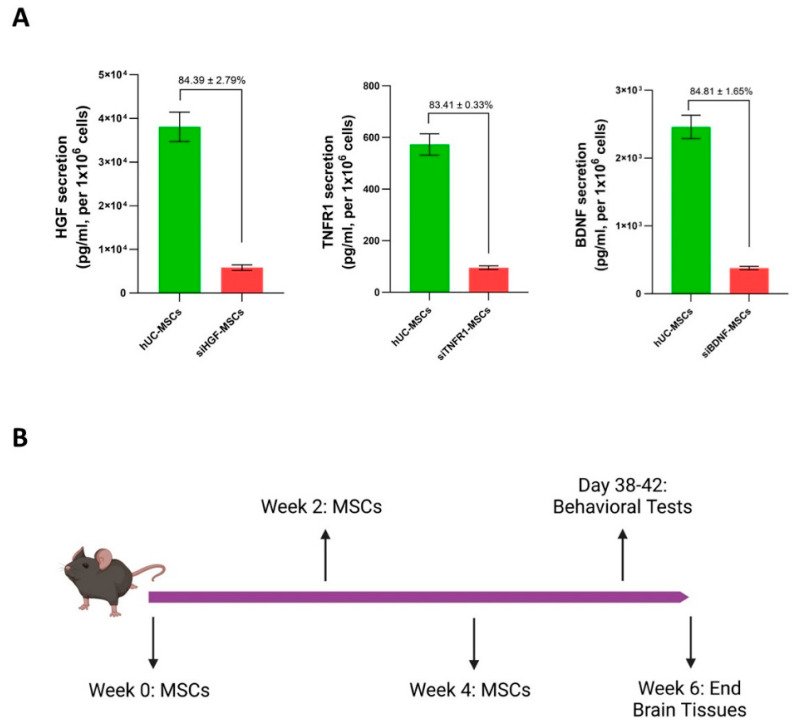
Protein production check and timeline of study. (**A**). Gene-silenced MSCs exhibited a reduction of over 80% in the release of HGF, TNFR1, or BDNF following siRNA transfection. 2 × 10^6^ hUC-MSCs were cultured in T175 flasks and incubated overnight. The following day, siHGF, siTNFR1, and siBDNF were separately transfected into the cells at a final concentration of 10 nM in serum-free medium. Six hours post transfection, the serum-free medium was replaced with complete medium. Forty-eight hours after transfection, the medium were harvested, and the concentration of HGF, TNFR1, and BDNF in both gene-silenced MSCs and control MSCs were measured by ELISA kits. The cell count in flasks were determined to adjust for protein production as follows: Production = 10^6^ × (concentration in total medium/cell number). The percentage of reduction was calculated using the formula Reduction % = 100 × (adjusted concentration in hUC-MSCs − adjusted concentration in gene silenced-MSCs)/adjusted concentration in hUC-MSCs. (**B**). Timeline of cell administration and tissue collection. The day of the initial dose of MSCs was designated as week 0. Cells were administered to mice biweekly. Behavioral tests began in the fifth week following the first dose. Immediately after tests, mice were euthanized to harvest brains, which occurred in the sixth week after the first dose.

The novel object recognition test was conducted 5 weeks after the first dose (days 38–39) (Figure 1). The test consisted of three phases: habituation, training, and memory retention. Each mouse was placed in a 25 × 25 × 25 cm box and allowed to explore for 5 min without any objects (habituation phase). During the training phase, two objects were placed in the back corners of the box. The mouse was then placed in the center of the box and allowed to explore for 3 min. The total time spent exploring the two objects was recorded. In the memory retention phase, 24 h after training, one of the familiar objects was replaced with a novel object. The mouse was allowed to explore for 3 min, and the time spent exploring each object was recorded. The novel object recognition index was calculated as follows:

$$Novel\;Object\;Recognition\;Index\;\left(\%\right)=100\;(Novel\;Object\;Exploring\;Time)/(Novel\;Object\;Exploring\;Time+\;Familiar\;Object\;Exploring\;Time)$$

A lower index indicates impaired cognitive function, while a higher index indicates normal cognitive function.

### 2.3. Morris Water Maze Test

**Figure 2:**
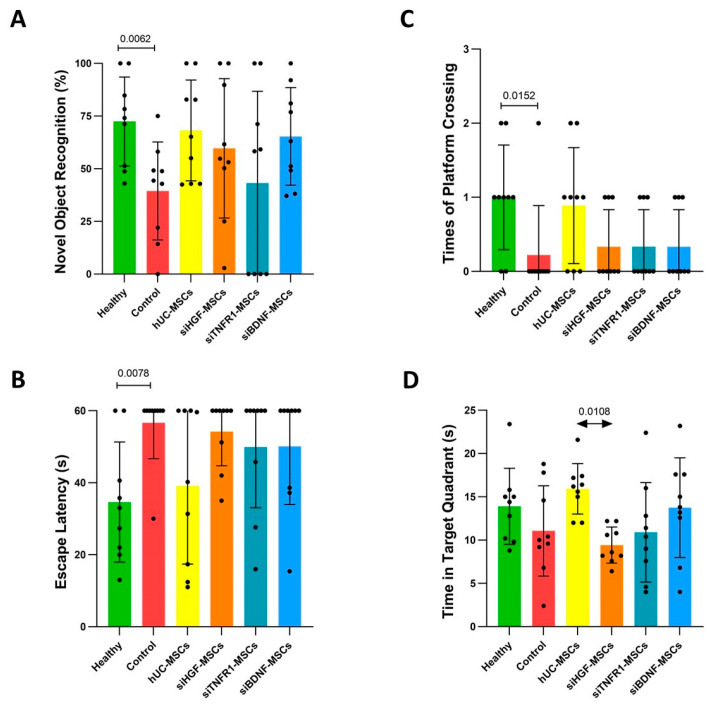
Behavioral tests results. (**A**). New object recognition. Healthy group–control group: unpaired *t*-test. Control–treatment groups and hUC-MSC group–gene-silenced groups: Kruskal–Wallis test and Dunn’s multiple comparison. (**B**,**C**). Escape latency and crossing platform assessments in Morris Water Maze. Healthy group–control group: Mann–Whitney test. Control–treatment groups and hUC-MSC group–gene-silenced groups: Kruskal–Wallis test and Dunn’s multiple comparison. (**D**). Time in target quadrants in Morris Water Maze. Healthy group–control group: unpaired *t*-test. Control–treatment groups and hUC-MSC group–gene-silenced groups: One-way ANOVA and Dunnett’s multiple comparison.

The Morris water maze test was conducted 5 weeks after the first dose (days 40–42) (Figure 2). The water maze apparatus consisted of a circular pool (120 cm in diameter, 50 cm in height) filled with water maintained at 25 °C and a depth of 30 cm. The test comprised two parts: the place navigation test and the spatial probe test. In the place navigation test, mice were trained to locate a hidden platform (10 cm in diameter, 30 cm in height, submerged 2.0 cm below the water surface) over 2–3 days, with two trials per day. The platform was placed in one of the four quadrants of the pool, and the mice were released from one of four starting positions (east, west, south, or north). If a mouse failed to find the platform within 60 s, it was guided to the platform and allowed to stay there for 10 s. The spatial probe test was conducted on the day after the final training session. The platform was removed, and the mouse was released from the quadrant opposite to where the platform had been located. The number of crossings over the platform’s previous location, escape latency, time spent in the target quadrant, and average swimming speed were recorded and analyzed using the Noldus EthoVision XT 17.0.

### 2.4. Detection of Brain Oxidative Stress and Inflammatory Cytokine Levels

At the end of the water maze test, the right hemisphere of each mouse’s brain was collected and homogenized in saline to prepare a 10% homogenate. The homogenate was centrifuged at 13,000 rpm for 10 min to obtain the supernatant. The superoxide dismutase (SOD, A001-3-2, Nanjing Jiancheng Bioengineering Institute, Nanjing, China) and malondialdehyde (MDA, A00s3-1-2, Nanjing Jiancheng Bioengineering Institute), inflammatory cytokines (IL-6, 302550-003, Invitrogen (Waltham, MA, USA); IL-1β, 282243-011, Invitrogen; TNF-α, 270856-021, Invitrogen) and AD-related proteins (Aβ40, KMB3481, Invitrogen; Tau, KMB7011, Invitrogen) were measured using ELISA kits.

### 2.5. MSCs Preparation

The hUC-MSCs were a gift from Beijing Baylx Biotech Co., Ltd. (Beijing, China). The characterization of MSCs through measurement of CD73, CD90, CD105, CD19, CD34, CD11b, CD45, and HLA-DR was performed by Beijing Baylx Biotech Co., Ltd. [19]. Prior to the experiment, cells were thawed in a 37 °C water bath and incubated with DMEM/F12 (Thermo Fisher, Waltham, MA, USA, Cat. 12500096) medium containing bFGF and 10% fetal bovine serum (Thermo Fisher) at 37 °C and 5% CO_2_. Once reaching 70–80% confluence, they were digested by StemPro^®^ Accutase Reagent (Thermo Fisher) for propagation. To generate gene-silenced MSCs, hUC-MSCs were seeded in a T175 flask and separately transfected by siHGF (Synbio-Tech, Suzhou, China; 5′-CCAAUGUGCUAAUAGAUGUdTdT-3′, 5′-ACAUCUAUUAGCACAUUGGdTdT-3′), siTNFR1 (Thermo Fisher, s14266), or siBDNF (Thermo Fisher, s1964) through lipofectamine RNAi-Max (13778150, Thermo Scientific). The knockdown efficiency of these siRNA was evaluated through western blot or RT-qPCR. The production of protein after transfection was confirmed by ELISA kits (DRT100, R&D Systems (Minneapolis, MN, USA); ab275901, Abcam (Cambridge, UK); ab212166, Abcam).

### 2.6. Statistical Analysis and Graphs

Data are presented as mean ± SD. Before analyzing differences, data were checked for distribution via the Shapiro–Wilk test. The healthy group and the control group were compared using either an unpaired *t*-test or a nonparametric test, with significant differences indicated by an H-shape. The control group and the treatment groups were compared through either one-way ANOVA or a nonparametric test, followed by multiple comparisons, with significant differences denoted by lines. The hUC-MSCs group and the gene-silenced groups were compared through either one-way ANOVA or a nonparametric test, followed by multiple comparisons, with significant differences indicated by arrows. All the analysis was performed using GraphPad Prism 9.0. Differences were considered statistically significant when *p* < 0.05, and the *p*-values of statistically significant results are reported on the graphs. Each black dot represents an animal on graph.

The graphical abstract and the image of the timeline were created with BioRender.com. Other images were created with GraphPad Prism 9.0.

## 3. Results

### 3.1. Effects of MSCs on Behavioral Performance in AD Mice

Before applying to animals, the production of HGF, TNFR1, and BDNF in hUC-MSCs was evaluated through ELISA kits. All siRNAs resulted in a reduction in production exceeding 80%, as indicated in Figure 1A. Prior to behavioral tests, APP/PS1 mice received three doses of either hUC-MSCs or gene-silenced MSCs, with the cells being administered biweekly. Five weeks after the first dose, the novel object recognition test and Morris water maze test were conducted sequentially. Following the water maze, animals were euthanized to harvest brain tissues. The timeline of the study is shown in Figure 1B.

In the novel object recognition test, compared to the healthy group, the control group showed significantly reduced exploration of the novel object (Figure 2A). The hUC-MSC group and siBDNF-MSC groups showed improvement in novel object exploration compared to the control group, although there were no significant differences between them (Figure 2A). The improvement was reduced in the siHGF-MSC and siTNFR1-MSC groups (Figure 2A). Notably, the performance of the siTNFR1-MSC group in the novel object recognition index was close to that of the control group.

In the Morris water maze test, compared to the healthy group, the control group exhibited significant memory and behavioral deficits, which were displayed as prolonged escape latency and reduced platform crossings (Figure 2B,C). The time spent in target quadrants also reduced, although this was not significant (Figure 2D). Compared to the control group, the hUC-MSC group showed significant improvements in escape latency and platform crossings. The time in the target quadrant increased, although this was not significant. Compared to the hUC-MSC group, the siHGF-MSC, siTNFR1-MSC, and siBDNF-MSC groups lost the improvement effects. A significant reduction of the time in the target quadrant was observed siHGF-MSC.

These results suggest that HGF, TNFR1, and BDNF are involved in the behavioral improvements mediated by MSCs in AD mice.

### 3.2. Effects of MSCs on Aβ40 and Tau Levels in AD Mouse Brain Tissue

**Figure 3:**
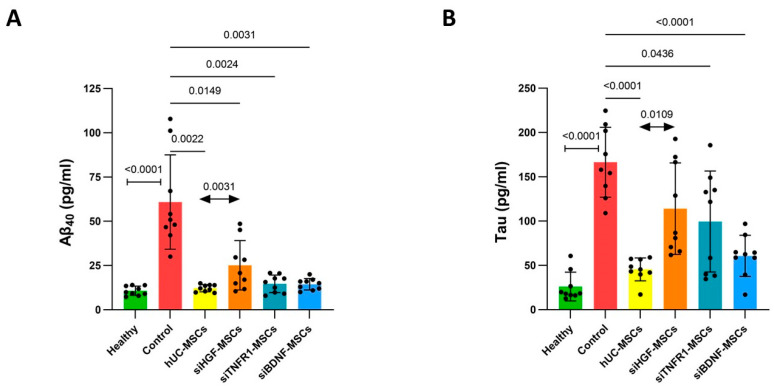
Total Aβ_40_ and Tau in the right brain right after behavioral tests. (**A**). Aβ_40_ levels in the right brain. Healthy group–control group: unpaired *t*-test. Control–treatment groups: Welch’s ANOVA test and Dunnett’s T3 multiple comparison. hUC-MSC group–gene-silenced groups: one-way ANOVA and Dunnett’s multiple comparison. (**B**). Tau levels in right brain. Healthy group–control group: Mann–Whitney test. Control–treatment groups and hUC-MSC group–gene-silenced groups: Welch’s ANOVA test and Dunnett’s T3 multiple comparison.

Aβ plaques and hyperphosphorylated Tau protein are hallmark pathological features of AD. Their accumulation in the hippocampus is related to memory decline. This accumulation is not limited to hippocampus; is can spread to multiple brain areas, including the temporal lobe, parietal lobe, and prefrontal cortex, ultimately resulting in cognitive decline, with Aβ driving the propagation of Tau pathology [20,21,22]. Therefore, we assessed the Aβ and Tau load across the entire brain. Compared to the healthy group, the model group showed significantly elevated levels of Aβ40 and Tau in brain homogenates (Figure 3). Compared to the model group, the hUC-MSC group and gene-silenced MSC groups showed significantly reduced Aβ40 levels (Figure 3A). The hUC-MSC, siTNFR1-MSC, and siBDNF-MSC groups showed significantly reduced Tau levels (Figure 3B), whereas the siHGF-MSC group showed reduced Tau, but the effect was not significant (Figure 3). Compared to the hUC-MSC group, the siTNFR1-MSC and siBDNF-MSC groups showed similar reduction effects on the Aβ40 level, suggesting that TNFR1 and BDNF may not be involved in MSC-mediated inhibition of Aβ40. However, the siHGF-MSC group showed slightly higher Aβ40 levels, which reached statistical significance, indicating that HGF may play a role in MSC-mediated inhibition of Aβ40. Additionally, Tau levels were more elevated in the gene-silenced groups compared to the hUC-MSC group (Figure 3B), especially in the siHGF-MSC group, where the difference reached statistical significance, indicating that HGF, TNFR1, and BDNF may be involved in MSC-mediated inhibition of Tau.

Overall, these results suggest that HGF may be more important for MSCs to repress Aβ40 and Tau.

### 3.3. Effects of MSCs on Neuroinflammation in AD Mice

**Figure 4:**
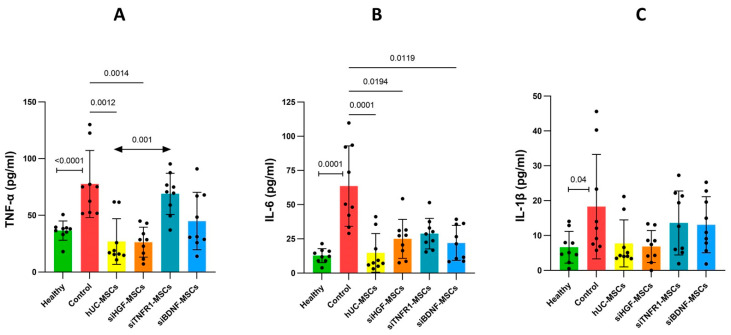
Inflammatory cytokines levels in the right brain right after behavioral tests. (**A**). TNF-α level. Healthy group–control group: Mann–Whitney test. Control–treatment groups and hUC-MSC group–gene-silenced groups: Kruskal–Wallis test and Dunn’s multiple comparison. (**B**). IL-6 level. Healthy group-control group: unpaired *t*-test. Control–treatment groups and hUC-MSC group–gene-silenced groups: Kruskal–Wallis test and Dunn’s multiple comparison. (**C**). IL-1β level. Healthy group-control group: Mann–Whitney test. Control–treatment groups: Kruskal–Wallis test and Dunn’s multiple comparison. hUC-MSC group–gene-silenced groups: one-way ANOVA and Dunnett’s multiple comparison.

The accumulation of Aβ and Tau can induce neuronal toxicity and prime microglia and astrocytes to inflammatory status [6,23]. Pathological microglia and astrocytes are widely distributed throughout the brain [24,25]. We hypothesized that the overall levels of inflammatory cytokines may increase as the disease progresses. By analysis of brain homogenate, we found the model group had significantly elevated levels of TNF-α, IL-6, and IL-1β, compared to the healthy group (Figure 4). Compared to the model group, hUC-MSC treatment significantly reduced TNF-α and IL-6 levels, while the IL-1β level was also reduced, but not significantly (Figure 4). These results suggest that hUC-MSCs have potential to suppress neuroinflammation. Compared to the model group and hUC-MSC group, the siTNFR1-MSCs group showed reduced efficacy in suppressing these cytokines (Figure 4), indicating that TNFR1 plays a role in MSC-mediated immune regulation. Notably, the siTNFR1-MSC group obviously lost the repression of TNF-α. In contrast, silencing HGF did not affect MSC-mediated suppression of TNF-α and IL-1β, although it resulted in a slight reduction in the suppression of IL-6 (Figure 4). This observation suggests that HGF may not play a significant role in the regulation of these cytokines. Furthermore, silencing BDNF resulted in a modest reduction in the repression of TNF-α. A comparable reduction in the repression of IL-1β was observed in comparison to siTNFR1-MSC (Figure 4). A mild and comparable reduction in the repression of IL-6 was found across all gene-silenced groups, when compared to the hUC-MSCs group. Similarly, an in vitro model demonstrates that overexpression of BDNF reduces the production of TNF-α,IL-6 and IL-1β in human nerve cells, in which p-38 is inhibited [18]. Additionally, BDNF pretreatment suppresses their levels in the cortex and hippocampus in a model of pneumococcal meningitis [26]. These findings suggest that BDNF may have anti-inflammatory ability.

In summary, TNFR1 and BDNF may be implicated in the regulation of neuroinflammation, whereas HGF appears to have a lesser role.

### 3.4. Effects of MSCs on Brain Oxidative Stress in AD Mice

**Figure 5:**
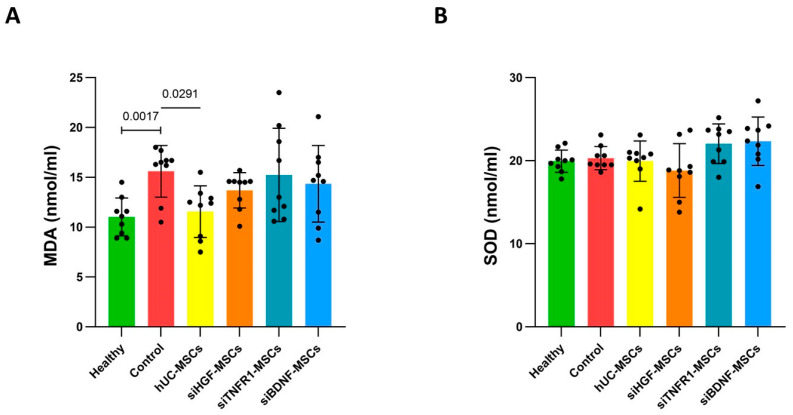
Oxidative stress assessments in the right brain right after behavioral tests. (**A**). Lipid peroxidation MDA level. Healthy group–control group: Mann–Whitney test. Control–treatment groups and hUC-MSC group–gene-silenced groups: Kruskal–Wallis test and Dunn’s multiple comparison. (**B**). Anti-oxidation enzyme SOD level. Healthy group–control group: unpaired *t*-test. Control–treatment groups: one-way ANOVA and Dunnett’s multiple comparison. hUC-MSC group–gene-silenced groups: Kruskal–Wallis test and Dunn’s multiple comparison.

In addition to neuroinflammation, Aβ deposition and Tau hyperphosphorylation can lead to mitochondrial dysfunction, resulting in the accumulation of free radicals and oxidative damage to lipids and other macromolecules [27]. Malondialdehyde (MDA) is a product of lipid peroxidation, and its levels reflect the extent of oxidative stress. Superoxide dismutase (SOD) is an important antioxidant enzyme that protects cells from oxidative damage. Compared to the healthy group, the model group showed significantly elevated MDA levels (Figure 5A), indicating increased oxidative stress in AD mice. hUC-MSC treatment significantly reduced MDA levels, suggesting that hUC-MSCs can alleviate lipid peroxidation (Figure 5A). In contrast, the siTNFR1-MSC, siHGF-MSC, and siBDNF-MSC groups showed higher MDA levels than the hUC-MSC group, with no significant differences compared to the model group (Figure 5A), indicating that gene silencing reduced the ability of MSCs to alleviate lipid peroxidation. Unlike MDA, SOD levels did not differ significantly among the healthy control, model control, and treatment groups (Figure 5B). However, it remains unclear whether the modifications and functions of SOD vary between these groups. The current result suggests that hUC-MSCs and gene-silenced MSCs may not affect SOD expression or stability in AD mice. Further research is necessary to ascertain whether hUC-MSCs mitigate lipid peroxidation by modulating SOD function.

## 4. Discussion

This study demonstrates that hUC-MSCs can improve cognition, reduce total Aβ40 and Tau levels, and alleviate neuroinflammation and lipid peroxidation in AD mice. Gene silencing of HGF, TNFR1, and BDNF reduces the therapeutic efficacy of MSCs, highlighting the importance of these factors in MSC-mediated therapy for AD. However, total Aβ40 and Tau cannot accurately reflect pathology in specific regions. To address this limitation, staining of Aβ plaques and p-Tau in different brain regions will be performed in the future studies.

The effects of the three cytokines on alleviating neuroinflammation was evaluated. Neuroinflammation can trigger and aggravate various neurodegenerative diseases, including AD, especially during aging [28]. Central nervous system (CNS) inflammation usually is caused by microglia and astrocytes [29], in which inflammatory astrocytes can be induced by active microglia [30]. Neuroinflammation results in elevation of hallmarks Aβ and Tau, which further lead to synaptic and neuronal loss [31]. Suppressing neuroinflammation can mitigate neurodegeneration, which could be achieved through strategies such as the overexpression of TREM2 [32]. This approach promotes anti-inflammatory microglia and inhibits the JAK/STAT/SOCS signaling pathway [32]. Additionally, it has been proven that the exosomes of various sources of MSCs can suppress neuroinflammation to alleviate AD, as evidenced by the reduced inflammatory microglia, lower inflammatory cytokine levels, or lesser activation of NLRP3 inflammasome [33,34,35]. Our hUC-MSCs not only reproduced the inhibition of inflammatory cytokines, but also reduced hallmarks Aβ and Tau, and promoted cognition. Moreover, we separately knocked down TNFR1, HGF, and BDNF to explore their roles in suppressing neuroinflammation. The soluble TNFR1 produced by TACE can neutralize TNF-α and inhibit inflammation [36,37,38]. In contrast, the transmembrane TNFR1 can activate various signaling pathways, including NFκB, MAPK, apoptosis, and necroptosis, through its cytoplasmic region [39], ultimately leading to cell death and inflammation in autoimmune diseases and central nervous system disorders [15]. Notably, in APP/PS1 mice, our siTNFR1-MSC group not only failed to repress TNF-α but also exhibited a partial loss of repression of IL-6 and IL-1β, indicating a trend towards diminished inflammation suppression. This finding aligns with our previous work, which demonstrated that the production of multiple chemokines, cytokines, or growth factors is compromised, and the ability to repress immune infiltration in vivo is impaired in siTNFR1-MSCs [16]. These results suggest that TNFR1 mediates anti-inflammation in hUC-MSCs. We hypothesize that silencing of TNFR1 not only stops the production of soluble TNFR1, but also diminishes the activation of NFκB and MAPK pathways in response to TNF-α, consequently leading to a reduced expression of immune regulatory genes in hUC-MSCs. The reduced translocation of NFκB p65 in the MSC nucleus after TNFR1 knockdown has been proven by Akaitz Dorronsoro et al. [40]. The observation of NFκB p65 location and the expression of its downstream genes in MSCs will be evaluated in our future studies. Moreover, the evaluation of microglia polarization and exploration of microglia cytokine expression after MSCs treatments will be performed. Unlike TNFR1, BDNF acts as a ligand that interacts with receptor TrkB and p75NTR, influencing various signaling cascades, including MAPK, PI3K, and NFκB [41]. BDNF plays an important role in neuronal regeneration, repair and protection, especially in the protection of the hippocampus [41]. Elevation of BDNF in hippocampus participates in improvement of cognition in AD models [42]. Furthermore, in a model of perioperative neurocognitive disorder (PND), hUC-MSCs inhibit neuroinflammation and promote upregulation of neural stem/progenitor cell markers in the hippocampus, partially through BDNF/TrkB/CREB signaling [43]. According to these findings, we hypothesize that the BDNF produced by hUC-MSCs may enhance the survival of neural stem/progenitor cells in the subgranular zone of the dentate gyrus and improve astrocytes in the APP/PS1 hippocampus, thereby indirectly mitigating inflammation and cognitive deficits. Observations of the status of neural stem/progenitor cells and astrocytes after treatments will be conducted in future. However, in addition to the benefits, there are also risks associated with BDNF. First, the polymorphism of BDNF, such as Val66Met, may be linked to elevated p-Tau and cognitive impairment [44]. Second, BDNF may increase the risk of epilepsy through interfering with glutamatergic synaptic transmission and GABAergic inhibition in neurodegenerative diseases [45,46]. Furthermore, since TrkB is expressed not only in the central nervous system but also in peripheral nervous systems, and given that TrkB can be upregulated due to pathological conditions, systemic delivery of BDNF may lead to overactivation of TrkB signaling, leading to excitotoxicity [47]. Therefore, monitoring BDNF concentrations following MSCs’ infusion is essential. Further clinical studies should consider subgroups based on the risk of epilepsy or genetic polymorphism.

Unlike TNFR1 and BDNF, HGF has lesser effects on TNF-α, IL-6 and IL-1β, but it displays more influence on Aβ40 and Tau. The relationship between HGF and Alzheimer’s disease has been studied. H Fenton et al. demonstrated increased HGF in AD patients’ brains, particularly within reactive astrocytes and microglia surrounding senile plaques [48]. Y Tsuboi et al. highlighted a significant association between cerebrospinal fluid (CSF) HGF levels and white matter hyperintensities (WMH) in AD patients. Findings from Zhao et al. revealed that elevated CSF HGF levels in patients with mild cognitive impairment (MCI) correlated with AD hallmarks (Aβ42, pTau, tTau) and accelerated cognitive decline [49]. These findings suggest that the increase of HGF may reflect neuroinflammation or white matter damage and HGF may serve as a dynamic indicator of disease progression. However, despite the increase of HGF, the decline of HGF receptor c-MET in the hippocampal pyramidal neurons was revealed in AD patients [50], which may cause reduction of HGF/c-MET signaling and cognitive impairment. The role of HGF in AD has been further elucidated by Takeuchi et al. through delivery of the human HGF gene into the cerebral ventricles of Aβ-infused mice [51]. The overexpression of human HGF upregulated both endogenous mouse HGF protein and c-Met mRNA levels [51]. Moreover, transfer of HGF alleviated cognitive impairment and oxidative stress, and enhanced angiogenesis and synaptophysin levels [51]. These findings suggest that HGF exerts neuroprotective effects. To date, many preclinical studies have demonstrated that HGF enhances synaptic plasticity and cognitive function, and modulates neuroinflammation, likely through the activation of the PI3K/AKT and MAPK signaling pathways [52]. Furthermore, according to findings from Yali Jia et al., hUC-MSCs may exert effects in the hippocampus through HGF-cMet-AKT-GSK3β signaling [14]. Based on these findings, we plan to investigate the changes in c-Met expression in the hippocampus, preferentially monitoring c-Met-expressing cells in that region, and observing synaptic pathology, in future studies. In addition, given that microglia play a crucial role in the clearance of Aβ and Tau, we will also focus on identifying protective subtypes of microglia, evaluating microglial phagocytosis, and detecting genes related to plaque clearance, such as TREM2, as part of our research plan.

In addition to individual pathways, HGF and BDNF signaling may interact synergistically. For instance, Hecht et al. revealed coexpression of TrkB with c-Met, resulting in the upregulation of HGF/c-MET in neuroblastomas [53]. In macrophages, HGF/c-MET induces phosphorylation of CREB via the PI3K pathway, shifting the phenotype towards an M2 like [54]. Additionally, a study on renal cancer revealed that HGF/c-Met enhances the level of nuclear factor E2-related factor 2 (Nrf2), thereby alleviating oxidative stress and apoptosis, both in vitro and in vivo [55]. Furthermore, a depression model suggests that Nrf2 acts as a transcription factor for BDNF [56]. Without Nrf2, the BDNF–TrkB axis was diminished [57]. Based on these findings, we hypothesize the existence of an HGF–Nrf2-BDNF axis that contributes to ROS clearance and neuronal survival.

The role of SOD in alleviating oxidative stress remains ambiguous in our study. Further experiments should assess whether hUC-MSCs enhance SOD enzymatic activity. Additionally, the mechanisms by which hUC-MSCs reduce oxidative stress require further investigation. It is important to determine whether hUC-MSCs regulate the Nrf2 pathway in APP/PS1 mice. Addressing these questions will refine the mechanistic understanding of mesenchymal stem cell (MSC) therapy in future studies.

The limitation of systemic delivery of MSCs is the pulmonary trap, which reduces the number of cells reaching the brain. Our findings indicate that a majority of hUC-MSCs remain in the lung within 24 h post-intravenous infusion, with a rapid decline in cell viability observed within 7 days post-infusion, consistent with previous report [58]. Repeated dosing, as employed in our study, is one strategy to mitigate this issue; however, it necessitates a large number of cells, and cell death diminishes overall efficiency. Hydrogel encapsulation may offer a solution, as this method has demonstrated improved MSC survival in vivo and maintenance of stemness [59,60]. Prolonging survival may reduce the number of cells required and enhance MSCs’ homing to the brain. Furthermore, hydrogel encapsulation is compatible with different delivery routes and engineered MSCs [61]. In addition to delivery route and survival improvement, gene polymorphism, such as BDNF as mentioned above, may influence therapeutic efficacy. When selecting MSCs donors, it is essential to consider critical genes and gene mutations. Cells carrying oncogenes, such as those with the p53 mutation, should be excluded. However, there is currently no consensus regarding the selection of donors based on specififc indications or the applicability of products to other diseases in relation to genetic mutations.

## Data Availability

The data presented in this study are available on request from the corresponding author.
